# Saliva, a bodily fluid with recognized and potential diagnostic applications

**DOI:** 10.1002/jssc.202100384

**Published:** 2021-08-18

**Authors:** Mozhgan Boroumand, Alessandra Olianas, Tiziana Cabras, Barbara Manconi, Daniela Fanni, Gavino Faa, Claudia Desiderio, Irene Messana, Massimo Castagnola

**Affiliations:** ^1^ Laboratorio di Proteomica, Centro Europeo di Ricerca sul Cervello IRCCS Fondazione Santa Lucia Roma Italy; ^2^ Dipartimento di Scienze Della Vita e Dell'Ambiente Università di Cagliari Cagliari Italy; ^3^ Dipartimento di Scienze Mediche e Sanità Pubblica, Sezione di Patologia Università di Cagliari, AOU of Cagliari Cagliari Italy; ^4^ Department of Biology, College of Science and Technology Temple University Philadelphia Pennsylvania USA; ^5^ Istituto di Scienze e Tecnologie Chimiche “Giulio Natta” (SCITEC) Consiglio Nazionale Delle Ricerche Roma Italy

**Keywords:** biomarkers, diagnostics, proteomics, saliva

## Abstract

Human whole saliva is a bodily fluid that can be obtained easily by noninvasive techniques. Specimens can be collected by the patient also at home in order to monitor health status and variations of several analytes of clinical interest. The contributions to whole saliva include secretions from salivary glands and, among others, from the gingival crevicular fluid that derives from the epithelial mucosa. Therefore, saliva is currently a relevant diagnostic fluid for many substances, including steroids, nonpeptide hormones, therapeutic drugs, and drugs of abuse. This review at first briefly describes the different contributions to whole saliva. A section illustrates the procedures for the collection, handling, and storage of salivary specimens. Another section describes the present use of whole saliva for diagnostic purposes and its specific utilization for the diagnosis of several local and systemic diseases. The final sections illustrate the future opportunities offered by various not conventional techniques with a focus on the most recent –omic investigations. It describes the various issues that have to be taken into account to avoid false positives and negatives, such as the strength of the experimental plan, the adequacy of the number of samples under study, and the proper choice of controls.

**Article Related Abbreviations**
ALS: amyotrophic lateral sclerosis
APCI: atmospheric pressure chemical ionization
aPRP: acidic proline‐rich protein
ATR‐FITR: attenuated total reflection‐Fourier transform infrared
bPRP: basic proline‐rich protein
CM: cutaneous subtype of Mastocytosis
COVID‐19: COronaVIrus Disease 19
CPSI‐MS: conductive polymer spray ionization mass spectrometry
DESI‐MSI: desorption electrospray ionization mass spectrometry imaging
gPRP: glycosylated (basic) proline‐rich protein
HOMD: Human Oral Microbiome Database
miRNA: microRNA
ML: machine learning
MS: multiple sclerosis
OSCC: oral squamous cell carcinoma
PCA: post‐conceptional age
pSS: primary Sjögren's syndrome
RT‐PCR: reverse transcriptase polymerase chain reaction
SARS‐CoV‐2: severe acute respiratory syndrome coronavirus 2
Sl: sublingual
Sm: submandibular
SM: systemic subtype of Mastocytosis
WS: whole saliva

## 1 INTRODUCTION

COVID‐19 pandemic has recently highlighted the utility and advantages of non‐invasive biological sample collection procedures for the screening of viral infections in a large population. Human saliva, due to the characteristics of its collection, immediately emerged as a challenging and suitable bodily fluid for this purpose [1, 2]. Indeed, whole saliva (WS) is a bodily fluid readily accessible, inexpensive, and stress‐free to collect. The composition of WS derives from the exocrine contribution of the three couples of major salivary glands and a variable number of minor salivary glands, in addition to a variety of non‐exocrine components, such as desquamated oral epithelial cells, leukocytes, micro‐organisms, and a serum‐like fluid emanating from the epithelial mucosa and gingival sulcus, this last called gingival (crevicular) fluid. Thanks to the mucosal and gingival fluid contribution, substances transported into the circulatory system are also present in WS. WS therefore has the full requirements for its use as a diagnostic fluid, and saliva test can allow, in particular cases (i.e., patients with difficult blood collection), to replace blood tests. This review, after a section discussing the procedure for collection, handling, and storage of salivary specimens, describes the present use of WS for diagnostic purposes. Subsequently, it illustrates the future opportunities offered by various techniques with a focus on the most recent –omic investigations.

## 2 SAMPLE VARIABILITY AND SPECIMEN COLLECTION

As any other bodily fluid utilized for diagnostic purposes, human WS requires proper procedures of collection, handling, and storage to preserve the integrity of potential biomarkers. Collection of saliva, although it is a simple procedure, must be done carefully, at first because WS is not a sterile bodily fluid. Endogenous and exogenous enzymes (from a complex and variable oral flora) with an unpredictable composition and activity are responsible for dynamic and continuous specimen modifications [3]. Moreover, the contribution of the different glands to WS changes according to circadian rhythms [4]. It is generally difficult or impossible to distinguish the contribution to WS of the submandibular (Sm) glands from that of the sublingual (Sl) glands because sublingual saliva empties either into the submandibular duct (Wharton's duct) via the sublingual duct (Bartholin's duct), or directly into the mouth via several small excretory ducts opening very near to the submandibular duct [4]. While Sm/Sl secretion predominates in the early morning, parotid secretion is at the maximum in the early afternoon. Therefore, salivary flow rate has its apex in the early afternoon (4 ± 2 PM) and it reaches a minimum during the night (4 ± 2 AM) [4]. Commonly, WS is collected in the morning, in order to have a similar contribution from parotid and Sm and Sl glands [5]. However, the time for collection may change according to the purpose of the study. For instance, if it is important to have a high concentration of proteins secreted specifically by the parotid glands, such as basic proline‐rich proteins (bPRPs), it is advisable to collect saliva in the early afternoon. Otherwise in the early hours of the morning in case the interest is focused on proteins mainly secreted by Sm and Sl glands, such as salivary cystatins (type S). Moreover, habits and/or problems of the donor, such as smoking, or hypo‐salivation should be taken into account [5]. Different collection procedures have been experimented, each one having its pros and cons. The most investigated collection procedures have been passive drooling, the use of cotton swabs, aspiration through soft devices positioned under the tongue (usually on the floor of the mouth, near to the frenulum), and chewing of paraffin gum. In passive drooling, the donor places her/him mouth over a test tube kept in ice bath, dropping saliva by gravity. It is more suitable for the collection of resting saliva, while the use of cotton swabs or other devices, as well as the chewing of paraffin gum, could promote secretion by mechanical stimulation and are more suitable for the collection of stimulated saliva. The use of swabs generates some bias, because salivary mucins are partly adsorbed on them, and in turn mucins adsorb several peptides and metabolites in an unpredictable way, altering the recovery of various components [6]. Furthermore, the decrease in viscosity facilitates enzymatic activities, particularly of proteolytic enzymes. Therefore, many studies have been devoted in the past to establish the best conditions to maintain the integrity of the sample during its collection and handling, with a particular concern to salivary proteins. Different studies [5, 7, 8] demonstrated that various protease inhibitors, such as ethylenediaminetetraacetic acid, 4‐(2‐aminoethyl)‐benzene‐sulfonyl fluoride hydrochloride, aprotinin, pancreatic trypsin inhibitor, leupeptin, antipain, and/or cocktail of them, were unable to adequately prevent proteolysis. Moreover, the presence of inhibitors increases the complexity of the sample and the use of peptides leads to interference in proteomic analyses. Cooling of the sample during collection (4°C) offered the chance to significantly reduce sample alteration, while surprisingly sample boiling did not prevent its integrity [5]. Regarding the stability of the sample, it must be emphasized that pH control plays a fundamental role. Indeed, acidification of the sample (pH 3 or less) completely abolished proteolysis, while in pH‐unadjusted salivary samples, proteins degraded completely in less than 4 h [5]. It has been evidenced that protein stability was already compromised at pH 4.0 and alkaline pH values, such as pH 10.0, only reduced the overall protein degradation [5, 7]. Acidification or freezing of the sample causes precipitation of several proteins, which can however be newly suspended for further analyses [9]. Whatever the method applied, the best way to avoid artifacts is to reduce, as much as possible, the time elapsing between collection and analysis, which in some cases can be less than 5 min. Alternatively, the sample has to be stored at −80°C (or less), because it has been evidenced that storage at −20°C did not prevent modifications [6]. The use of particular devices allows to collect selected glandular saliva: parotid saliva can be collected by a Lashley cup [10], and submandibular/sublingual saliva by means of an appliance described by Truelove *et al*. [11]. However, the use of these devices is invasive and requires the intervention of expert personnel. Therefore, in this review only the diagnostic use of WS will be reported.

## 3 CURRENT DIAGNOSTIC APPLICATIONS OF HUMAN WHOLE SALIVA

**FIGURE 1:**
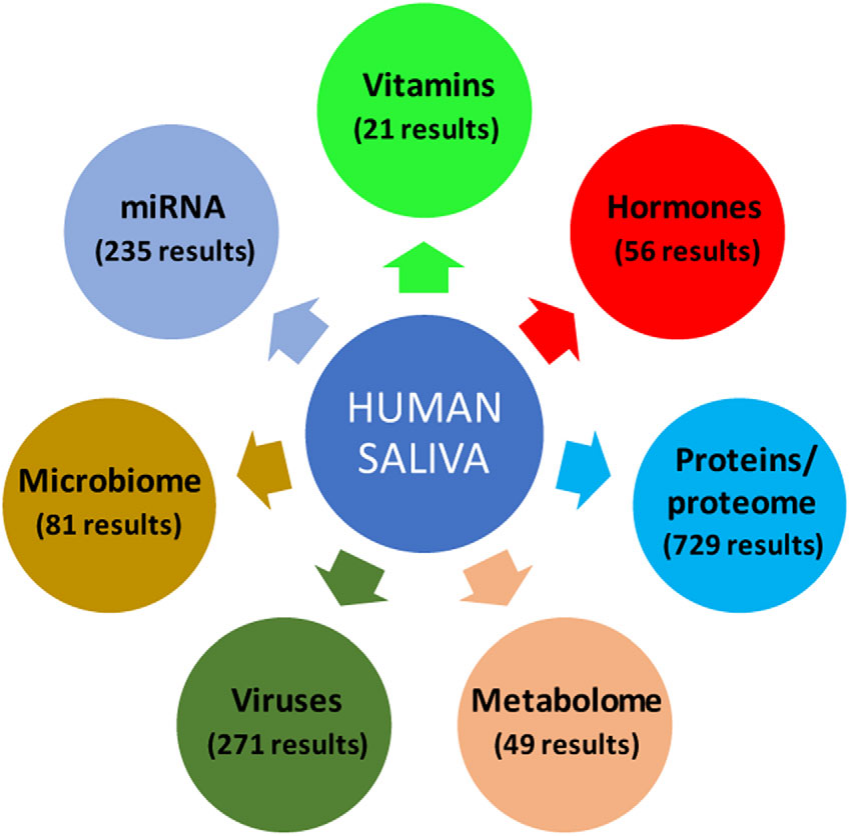
Scheme summarizing the main research fields in salivary diagnostic. Under each entry, the number of publications found on PubMed (https://pubmed.ncbi.nlm.nih.gov/advanced/), limited to the past 10 years, is reported

Human saliva is already utilized for diagnostic purposes (Figure 1). One of the first diagnostic applications of saliva concerned the measurement of salivary cortisol for the differential diagnosis of Cushing's syndrome, and to monitor the state of oxidative stress [12]. Recently, the determination of salivary cortisol in Cushing's syndrome has been implemented with the co‐determination of cortisone, its major metabolite with a significant complementary application for diagnostic purposes [13]. However, salivary cortisol determination, carried out during the dexamethasone suppression test for the diagnosis of subclinical hypercortisolism in adrenal incidentalomas, did not show the same accuracy of serum cortisol determination, as reported for diagnosis of the overt Cushing's syndrome [14].

Gao and colleagues [15] developed a method for the simultaneous quantitative analysis of estradiol and six other steroid hormones. Saliva was analyzed by HPLC‐atmospheric pressure chemical ionization (APCI) MS/MS coupled online with SPE. The protocol allowed the simultaneous determination of estradiol, cortisol, cortisone, testosterone, progesterone, corticosterone, and dehydro‐epiandrosterone in 100 μL volume of saliva with a limit of quantification of 5 pg/mL. The method showed sensitivity, linearity, and specificity, and a better performance with respect to immunoassay methods on the same samples (*n* = 16). Recently, a UHPLC–MS/MS method with ESI for the determination of testosterone in plasma and WS was developed [16]. The method used d3‐testosterone as an internal standard and was validated according to U.S. Food and Drug Administration guidelines.

Saliva is a suitable source for melatonin detection and quantification: beyond the well‐known chronobiological role, numerous additional functions have been recently identified; daytime saliva melatonin levels were analyzed from 108 young adults with ELISA in relation to expression levels of 72 inflammatory markers related to anxiety disorder or depression. The authors evidenced a positive correlation between daytime postprandial melatonin levels and CCL2/MCP‐1, CCL3/MPI‐1α, and VEGF‐A [17].

Other than hormones, WS is a suitable bodily fluid for the analysis of vitamins: in order to detect vitamin D deficiency, Clarke and colleagues [18] developed an HPLC‐MS/MS method to quantify 25‐hydroxy vitamin D in saliva using the deuterated compound as internal standard. The authors demonstrated that average values resulting from saliva collected on three consecutive days correlated with serum values, after adjusting for differences in saliva flow rate [18].

Human saliva is today also largely utilized for the detection of drugs of abuse. In some cases, it is possible to utilize small portable devices for on‐site sample collection and analysis to allow a rapid test on suspected individuals [19]. These devices consist of a saliva collector and a built‐detection system (usually an immunoassay test strip) for the screening of multiple drugs or drug classes. The presence of the drug (or drug class) in the sample can be evidenced by the appearance of a line on the device, or better by reading on the strip the color intensity, proportional to drug concentration [20]. Although modern devices demonstrated satisfactory sensitivity, specificity, and efficiency, they are still strongly dependent on the type of analyte, the time of collection, and the presence of contaminants [21]. Obviously, the presence of the drug of abuse has to be confirmed by more strong laboratory analyses. The immunoassay screenings, characterized by low cost and speed of analysis, have several drawbacks, thus the current analytical gold standard is represented by methods based on HPLC‐MS/MS. In this way, it is possible to use oral fluid for the detection of amphetamines, cannabis derivatives, cocaine, opioids, and benzodiazepines, and this issue has been recently reviewed [19]. HPLC‐MS/MS techniques are also able to identify new psychoactive substances, and WS offers some advantages over urine and/or blood monitoring, thanks to its non‐invasive and fast collection. Moreover, urine collection is also affected by the risk of specimen adulteration [22].

As previously described, human saliva is in continuous contact with oral epithelia, which are colonized by a variable and not fully characterized oral flora. It is now clearly established that the gastrointestinal tract is populated by a variegated microflora, whose number of cells is comparable and sometimes exceeds the number of somatic cells [23, 24].

The beneficial effect of this army of symbionts on human health has been largely demonstrated [25, 26]. The colonization of the gastrointestinal tract by the oral microbiota is common and therefore its composition could have a deep impact on the gastrointestinal microbiome in health and disease. The imbalance of the oral microflora can lead to the onset of various oral diseases, such as dental caries and periodontitis [27, 28], as well as systemic disease, such as gastrointestinal and nervous systemic diseases [29, 30, 31].

The Human Oral Microbiome Database (HOMD) is the first curated description of human‐associated oral microbiome with the purpose to clarify its role in health and disease [32]. Centered on a list of 16S rRNA gene‐based provisional naming schemes, HOMD reports the sequence of over 600 16S RNA gene libraries, and it links sequence data with phenotypic, phylogenetic, clinical, and bibliographic information. In the HOMD list, more than 700 microbial species of approximately 150 genera are reported. For example, genomes for 30 oral taxa and 202 strains of *Streptococcus* are available.

With this variegate population of host genera, it is not surprising that human saliva has been submitted to microbiological analyses carried out to detect bacteria, fungi, archaea, protists, and viruses in a lot of infectious diseases [26]. For instance, the evaluation of *Candida albicans* in saliva has been a common assay to distinguish oral candidiasis from healthy carriages [33]. In last years, human saliva has been utilized for the detection of Zika virus [34], cytomegalovirus [35], and papillomavirus infections too [36].

As already pointed out at the beginning of the review, the devastating world COVID‐19 pandemic has sparked interest in the development of screening tests with minimal impact on the patients. Indeed, the first test developed for COVID‐19 uses 15 cm swab cones in order to sample mucosal fluid deeply into the nasopharynx, on which to search for the presence of the virus by PCR analysis. The procedure has sensitivity and specificity of 95%, but sampling is uncomfortable, and it puts medical personnel at risk of contracting the virus, because requires close contact with patients. The need for an easier and safer sampling led researchers to look for alternatives and saliva could represent a valid alternative in COVID‐19 diagnosis [37, 38]. People being tested may simply drool into a bar‐coded plastic tube, seal it, and drop it into an envelope to be sent to a lab for the following analysis [39].

Recently, a fast test based on a customized sandwich lateral flow technique has been developed to detect the presence of SARS‐CoV‐2 in salivary samples using a polyclonal antibody directed against the viral spike protein [40]. The test validated on 122 patients showed a sensitivity of 92%, which is near to the performance of the nasal swab. A recent research showed better sensitivity of salivary samples, compared to nasopharyngeal or nasal swabs for the RT‐PCR analysis of asymptomatic and mild COVID‐19 infections [2]. Table 1 reports pros and cons of the main analytical methods used for the discovery of salivary biomarkers.

**TABLE 1:** Examples of analytical methods used in laboratory analysis of saliva and for the discovery of salivary biomarkers

Analytical method	Biomarker	Pros	Cons^a^	Reference
High‐Performance Liquid Chromatography (HPLC) coupled to Mass Spectrometry (MS)	Proteins Peptides Metabolites Hormones Vitamins Drugs of abuse	Simultaneous measurements of different analytes High analytical specificity High sensitivity at low concentration ranges Suitable for biomarker discovery	Expensive instrument Highly qualified personnel	[15, 16, 18, 19, 41, 42, 43, 44, 45, 46, 47, 48, 49, 50, 51, 52, 53]
Enzyme‐Linked Immunosorbent Assay (ELISA)	IgA Metabolites Proteins Peptides Virus	High sensitivity Low‐cost Speed analysis	Possible cross‐reactivity Measurement of single analytes Analytical specificity dependent on antibody performance Cannot discriminate between different proteoforms of the same biomarker	[17, 19, 45, 54, 46]
Conductive Polymer Spray Ionization Mass Spectrometry (CPSI‐MS)	Metabolites Drugs of abuse	Wide coverage of chemical species Low‐cost Speed analysis	Expensive instrument Highly qualified personnel	[55]
Two‐dimensional gel electrophoresis (2DE) coupled to mass spectrometry (MS)	Complex protein mixtures	Large number of proteins resolved in one analysis Rapid assessment of proteome variation between samples Suitable for large protein analysis Suitable for biomarker discovery	Low reproducibility Time consuming pre‐analytical steps Difficult to separate hydrophobic and extremely acidic or basic proteins Low dynamic range of proteins Low throughput and labor‐ intensiveness Expert personnel	[56]
Matrix‐Assisted Laser Desorption/Ionization‐Time‐of‐ Flight mass spectrometry (MALDI‐TOF MS)	Peptides/proteins microorganisms	Small amount of sample Speed analysis Suitable for biomarker discovery Simultaneous detection of multiple biomarkers	Cannot detect small peptides Results strongly dependent on the matrix type	[57]
Raman Spectroscopy (RS)	All biological molecules	Simultaneous detection of macromolecules Rapid Possible automation	Low sensitivity Sophisticated data analysis It is almost impossible to establish the identity (or structure) of biomarkers	[58, 59]
PCR‐based	Nucleic acids Virus Bacteria	Sensitive High analytical specificity Reproducible	Requires intact RNA	[2, 34–40, 60–63]
Electric Field‐Induced Release and Measurement (EFIRM)	Circulating single‐stranded DNA molecules and RNA	Rapid Sensitive Quali/quantitative analysis Easily automatable	Measurement of single analytes	[64]
Attenuated Total Reflection‐Fourier Transform Infrared (ATR‐FTIR) spectroscopy	Proteins, Lipids, Nucleic acids, Carbohydrates	Minimal or no sample preparation Non destructive Rapid spectrum recording	Selective partitioning of samples due to the hydrophobic nature of the ATR prism Error comparing samples with different refractive indices	[65]
Nuclear Magnetic Resonance (^1^H‐NMR) spectroscopy.	Metabolites	Non destructive Easy sample preparation	Expensive instrument Expert personnel	[66]
Electrochemiluminescence (ECL) biosensors	Metabolites Proteins Nucleic acids metal ions	Sensitive Specific User‐friendly Rapid and robust Equipment‐free Deliverable to end‐users	Sensitive to sample matrix effects Low shelf life	[67, 68, 69, 70, 71]

^a^ All the analytical method suffered from variation in salivary flow rate within and between individuals which can impair the establishment of standard values [5].

## 4 THE APPLICATION OF –OMIC PLATFORMS TO HIGHLIGHT POTENTIAL SALIVARY BIOMARKERS

The applications above reported demonstrate that the use of WS as a diagnostic bodily fluid has been consolidated by numerous studies and salivary analyses are to date routine in many analytical laboratories. In the last 20 years, numerous efforts have been dedicated to the characterization of the set of proteins present in saliva, the salivary proteome, with the aim of using this knowledge for diagnostic purposes. However, as detailed in the following, the intra‐ and inter‐individual variations originating from the different contribution of the secretory sources, the biological rhythms, and the very high number of possible polymorphisms require a strong validation of the potential salivary biomarker before it can be confidently introduced into the clinical practice.

Without any doubt, ‐omic sciences, with their ability to simultaneously highlight a considerable number of analytes (proteins, nucleic acids, or metabolites), offer the possibility of amplifying the diagnostic potential of human saliva.

**FIGURE 2:**
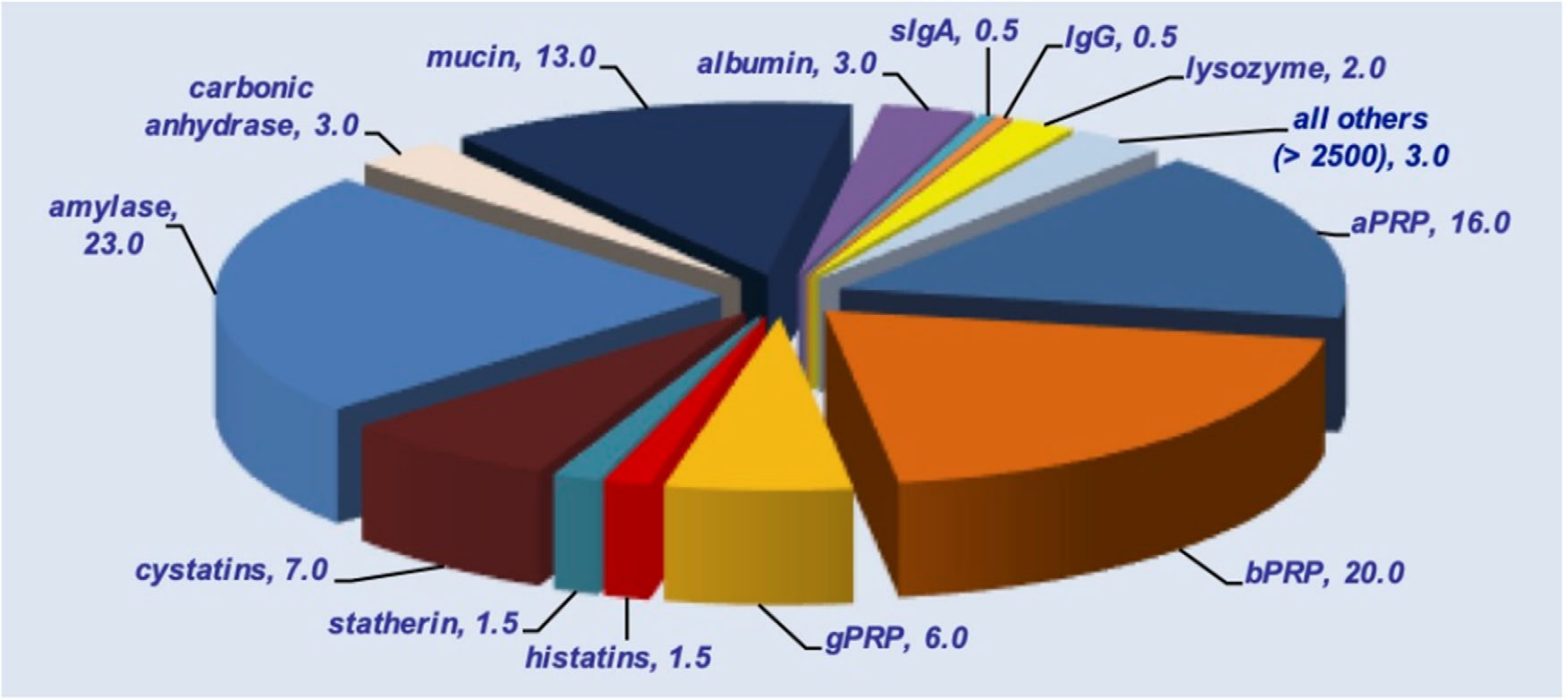
The figure shows the approximate contributions (% w) of the different protein families to whole saliva, assuming a similar involvement of parotid and Sm/Sl glands to the whole and a small involvement of the other sources listed in Table 2. For abbreviations see the legend of Table 2

For instance, shotgun proteomics has been able to detect more than 3000 salivary proteins, some not of human origin [8]. As shown in Figure 2, the main contribution to the composition of the human salivary proteome derives from a few protein families with approximately 500 components representing more than 95% (w/w) of all salivary proteins. All other proteins (more than 2500) account for less than 5% (w/w). Table 2 shows the main sources from which the most abundant protein families present in human saliva are derived, with the approximate contribution. Among the proteins specific to saliva (secreted by the salivary glands), the family of proline‐rich proteins (PRP) is the most abundant and it is divided into acidic proline‐rich proteins (aPRPs), basic proline‐rich proteins (bPRPs), and glycosylated (basic) proline‐rich proteins (gPRPs). The family is highly polymorphic: aPRPs are coded by two poly‐allelic loci, *PRH1* and *PRH2* the first with three alleles and the second with two alleles that overall can 18 different phenotypes, with different frequency [6].

**TABLE 2:** Approximate contribution of the most abundant protein families to the composition of human whole saliva

Peptide or protein family	Oral sources				
Peptide or family	Parotid	Sm/Sl	Minor salivary glands	Epithelial exudate	GCF
aPRPs	****	**	*		
bPRPs	****		*		
gPRPs	****				
Amylase	****	**	*		
Histatin 1	****	***	*		
Histatin 3	****	***			
Statherin	****	***			
P‐B peptide	**	****	*		
“S‐type” cystatins	*	****	*		
other cystatins	*	**	*		
MG1		***	**		
MG2		***	**		
HSA				**	**
IgG				**	
sIgA	*	*	*	*	
Lysozyme	*	*	*	*	*
α‐defensins				*	**
Thymosin β_4_				*	**
Thymosin β_10_				*	**

The table reports the approximate involvement of the main oral sources to the most abundant protein families detectable in human whole saliva.

Data on minor salivary glands are from Siqueira et al. [72]. Other data are mainly from Messana et al. [6] and from Ekström et al. [4]. Abbreviations: aPRPs (acidic proline‐rich proteins); bPRPs (basic proline‐rich proteins); gPRPs (glycosylated proline‐rich proteins): sIgA (secretory immunoglobulin A); IgG (immunoglobulin G); MG‐1 (large gel‐forming mucins, which include very large glycoproteins encoded by MUC5B, MUC4 and MUC19 genes); MG‐2 (small soluble protein encoded by the MUC7 gene); HAS (human serum albumin); Sm/Sl (submandibular/sublingual glands); GCF (gingival crevicular fluid). “S‐type” cystatins include cystatin S, S1, S2, SN, and SA. Other cystatins include cystatins A, B, C, D [73]. The large polymorphisms characterizing the proline‐rich protein family and other salivary protein families have been described in Oppenheim et al. [74], Manconi et al. [75], and Padiglia et al. [76]. P‐B peptide is also known as submaxillary gland androgen‐regulated protein 3B precursor (Swiss prot‐code P02814).

Analogously, bPRPs and gPRPs are coded by four loci, called from *PRB1* to *PRB4*. The *PRP2* and *PRP4* genes are three allelic loci existing as small (S), medium (M), and large (L). *PRB1* and *PRB3* genes are four allelic loci existing as small (S), medium (M), large (L), and very large (VL). Considering the possible homozygosity and heterozygosity they can generate at least 3600 phenotypes. This huge polymorphism of PRPs is further complicated by pre‐secretory cleavages of the pre‐pro‐proteins, (for *PRB1, PRB2, PRB4* loci) generating smaller peptides, by the existence of SNPs coding for variants (for *PRH2* and *PRB1‐4* loci) and splicing variants (for *PRB1* locus). Detailed descriptions of all these possibilities are reported in several recent references [75, 76, 77].

Other salivary protein families and polymorphisms have been described in Oppenheim et al. [74] and Manconi et al. [73]. Data on the proteome of minor salivary glands can be found in Siqueira et al. [72]. Other data are mainly from Messana et al. [6] and from Ekström et al. [4]. This large protein inventory may be the basis for comparative analyses conceived to characterize potential biomarkers of various oral and systemic diseases. However, there is no single proteomic strategy capable of characterizing the entire saliva proteome and indeed many studies on the same disease have been carried out with different experimental platforms. It is therefore not surprising to observe that different biomarkers for the same pathology have been proposed in different studies. Unfortunately, different biomarkers are sometimes suggested for the same pathology even when similar platforms are applied, and this generates legitimate doubts about the strength of the experimental plan, the adequacy of the number of samples under study, and the choice of controls [78]. Moreover, in proteomic platforms, the increased number of components under observation, strongly enhances the detection of variations connected to an interindividual polymorphism and not to a disease and, as above reported, human saliva is a bodily fluid displaying relevant polymorphisms [73, 74, 75, 76, 77]. As an example, proteomic analysis of saliva has been extensively utilized to reveal biomarkers of Sjögren's syndrome, suggesting a panel of proteins all linked to the inflammatory phase and these results have been largely reviewed [79, 80]. However, several studies carried out with different proteomic platforms in other diseases have often generated very different results. In order to avoid a report of non‐reproducible results, it is important to follow some rules: to carry out the analysis on an adequate number of samples in order to ensure the strength of the statistical differences, to follow strictly identical experimental protocols for different groups of patients, and to analyze the samples in random order. The use of ELISA methods for validation of proteomic results is also under discussion [54], because the antibody utilised may not have the proper selectivity to discriminate between the different proteoforms considered as potential biomarkers of the pathology.

One of the most investigated diseases to highlight salivary biomarkers by proteomic platforms is the oral squamous cell carcinoma (OSCC), which represents more than 90% of oral cancers. Many patients are today diagnosed with late state tumors, poor prognosis, and low survival rate. The availability of biomarkers that allow for the earliest possible diagnosis of OSCC is therefore an urgent need for clinicians. Many proteomics studies have been devoted to the search for early salivary biomarkers of OSCC, and the topic has been recently reviewed [81, 82]. Potential biomarkers identified in different studies were interleukins 6, 8, 1b, cyclin D1, and thioredoxin [41], resistin [42], S100A8 [43], α1‐antitrypsin, haptoglobin β chain, complement C3, haemopexin, and transthyretin [56], α1‐antitrypsin, complement C3, 4B, factor B, and leucine‐rich α−2‐glycoprotein [44], salivary cytokines [82], metalloproteinase‐1 [45], and complement factor H, fibrinogen alpha chain, and α1‐antitrypsin [46], to cite a few. Some of these studies agreed on the use of α1‐antitrypsin and some complement components as OSCC candidate biomarkers, but to our knowledge, none of these biomarkers have so far been transferred into clinical practice for the early diagnosis of OSCC.

Classical proteomic bottom‐up or shotgun platforms can be implemented by the results of the top‐down strategy, which focuses on the analysis of the intact proteome. Even if top‐down proteomic platforms allow detecting different proteoforms of each protein, they did not permit analyzing the same high number of proteins than bottom‐up proteomic platforms. Indeed, several high molecular weight proteins are inaccessible to top‐down analyses because most of them are not soluble under the acidic conditions normally used for MS and mass spectrometers can analyze only proteins whose dimensions do not exceed defined limits. These limitations have been partially overcome by top‐down analysis on Orbitrap platforms operating in Intact Protein Mode with trapping gas pressure set to 0.2 [83].

Moreover, the heterogeneity of some proteins, such as glycosylated proteins, generates crowded ESI spectra, which are not resolvable by the deconvolution software [54].

Despite these limitations, top‐down platforms applied to human WS have provided suggestions on molecular mechanisms at the basis of several pathologies. For instance, a study in patients with schizophrenia (n = 32) and bipolar disorder (n = 17) revealed more than 10‐fold increase in salivary levels of α‐defensins 1–4, S100A12, cystatin A, and S‐cysteinylated and S‐glutathionylated proteoforms of cystatin B compared to healthy non‐smokers (n = 19) and smokers (n = 12) control groups, suggesting dysregulation of the peripheral white blood cell immune pathway associated with schizophrenia [47].

In patients with Wilson's disease (n = 32), a rare inherited disorder of copper metabolism, manifesting hepatic, neurological, and psychiatric symptoms, top‐down analyses of saliva revealed significantly higher levels of S100A9 and S100A8, and their oxidized proteoforms with respect to controls (n = 32). Oxidation, occurring on methionine and tryptophan residues, and on the unique cysteine residue at position 42 in S100A8, and 3 in S100A9, generated glutathionylated, cysteinylated, sulfinic, sulfonic, and disulfide dimeric derivatives. Furthermore, saliva of Wilson's disease patients was characterized by high levels of two newly detected fragments of the polymeric immunoglobulin receptor, and of α‐defensins 2 and 4. Overall, the salivary proteome of Wilson's disease patients reflected the oxidative stress and inflammatory conditions characteristic of the pathology, highlighting differences that could be useful clues of disease exacerbation [48]. The top‐down analysis of saliva of patients with Down syndrome (n = 36) highlighted, in comparison with controls (n = 36), significantly increased salivary levels of S100A7, S100A8, and S100A12 [49]. A severe impairment of the repertoire of peptides involved in the protection of the oral cavity was found in children with type 1 diabetes, compared to age‐matched healthy controls. In particular, the increased levels of several proteolytic fragments of P‐C peptide in diabetics indicate a possible variation of exogenous proteinases in their oral cavity [50]. Multiple sclerosis (MS) is a chronic disease of the central nervous system characterized by inflammation, demyelination, and neurodegeneration of undetermined origin. To date, a single diagnostic test of MS does not exist and novel biomarkers are demanded for a more accurate and early diagnosis of the disease. A recent top‐down proteomic study investigated the salivary proteome of 49 MS patients and 54 healthy controls quantifying 119 salivary peptides/proteins. Statistical analysis revealed different levels of 23 proteins between the two groups; out of these, 8 proteins, namely mono‐ and di‐oxidized cystatin SN, mono‐ and di‐oxidized cystatin S1, mono‐oxidized cystatin SA and mono‐phosphorylated statherin, showed lower levels in patients with sclerosis multiple than the controls. The remaining 15 proteins, namely antileukoproteinase, two proteoforms of Prolactin‐Inducible Protein, P‐C peptide (Fr.1−14, Fr. 26−44, and Fr. 36−44), SV1 fragment of statherin, cystatin SN Des1−4, cystatin SN P11 → L variant, and cystatin A T96 → M variant, showed higher levels in MS patients than the controls. The differences observed between the salivary proteomic profile of patients suffering from MS and healthy controls are consistent with the inflammatory condition and altered immune response typical of the pathology [51].

Mastocytosis is a myeloproliferative neoplasm causing abnormal clonal mast cell accumulation in different tissues, such as skin and bone marrow. The cutaneous subtype (CM) is distinguished from the systemic subtype (SM). Moreover, SM patients can be grouped into SM with (SM+C) or without (SM‐C) additional cutaneous lesions, and their classification is often challenging. The study by Serrao et al. aimed to highlight variations in the salivary proteome of patients with different mastocytosis subtypes compared to healthy controls. The top‐down proteomics approach coupled to a label‐free quantitation revealed different salivary profiles in patients compared to controls, highlighting in particular, a downregulation of peptides/proteins involved in homeostasis and defense of the oral cavity, such as statherin, histatins, and acidic proline‐rich proteins (aPRPs), as well as in innate immunity and inflammation, such as the cathepsin inhibitors, suggesting a systemic condition associated with an exacerbated inflammatory state [52].

The upregulation of antileukoproteinase and S100A8 suggested a protective role against the disease status. The two SM forms were distinguished by the lower levels of truncated forms of aPRPs, statherin, P‐B peptide, and cystatin D and the higher levels of thymosin β4 and α‐defensins 1 and 4 in SM‐C patients with respect to SM+C [52]. However, no early disease‐specific biomarkers revealed by top‐down platforms have so far been transferred into clinical practice.

**FIGURE 3:**
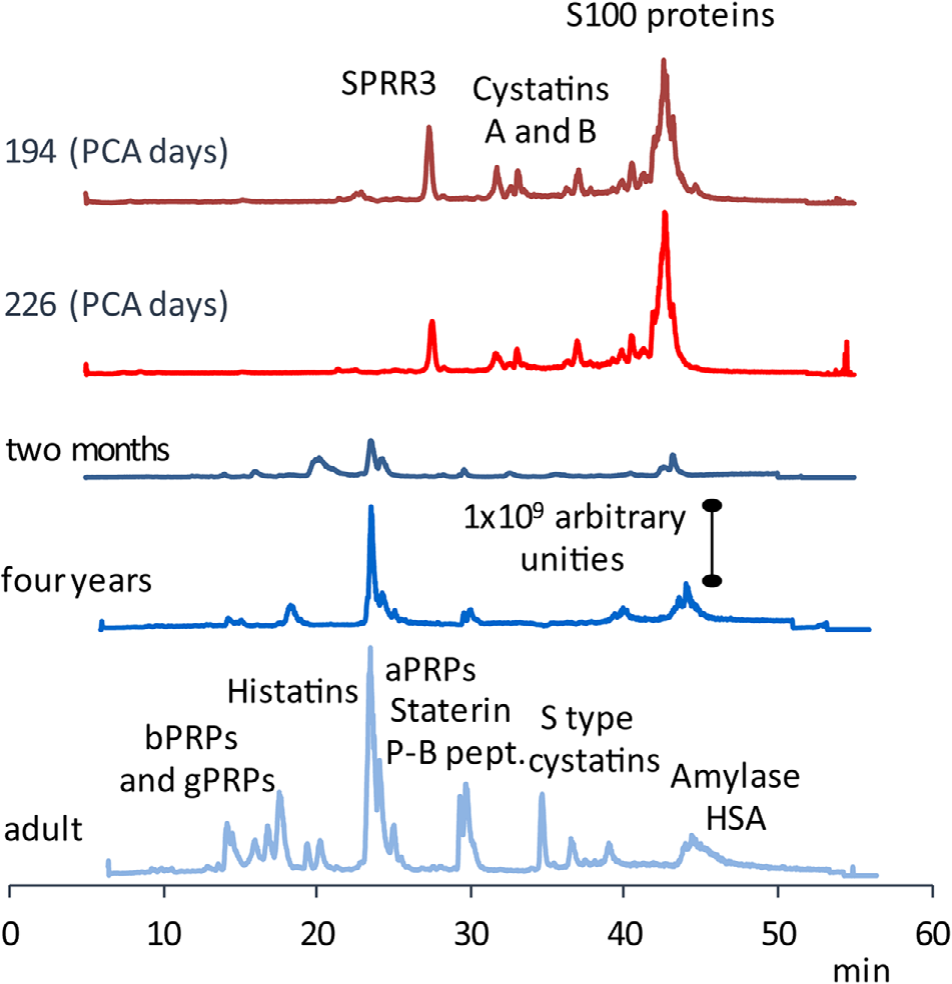
Changes of the typical HPLC‐ESI‐TIC (total ion current) profile of human saliva with the age, starting from preterm newborns (194 days of PCA, the age of this donor) up to adults. In preterm newborn saliva many proteins belonging to the cystatin family and S100 proteins are detectable. The proteins show a decrease in their level in the last period of fetal life and become almost undetectable in at‐term newborns. The profile typical of adults is commonly observed after the puberty [85]. SPRR3: small proline‐rich proteins 3 (Swiss prot code A5YKK8). For other abbreviations see Table 2

The collection of salivary samples is a routine procedure performed by the personnel of Neonatology Intensive Care Unit for the well‐being and survival of preterm newborns, in order to avoid its pouring into the lungs, and the sample is usually discharged. Its use is therefore ethically acceptable. The investigation by a top‐down platform of the oral fluid of preterm newborns surprisingly disclosed a different proteome with respect to that of adults [84]. Indeed, preterm newborn salivary proteome is characterized by elevated levels of more than 40 proteins, the majority pertaining to the S100, cystatins A and B, and thymosin's families, usually not detected or revealed at very low levels in adult saliva (Figure 3). Levels of these proteins decrease according to the post‐conceptional age (PCA) and when premature infants reach a PCA of approximately 280 days, corresponding to the normal term of delivery, composition of saliva becomes similar to that of at‐term newborns at birth. In this way, analysis of saliva allowed to investigate proteins active during fetal growth. All the proteins characterized in the oral fluid of preterm newborns have indeed relevant roles in the angiogenesis and in tissue modeling. Moreover, the different proteoforms of the same protein detectable in preterm newborn saliva allowed to establish that the activities of several convertases and exopeptidases are higher in the preterm newborns than in the adult [84]. On the contrary, the Fam20C kinase, responsible for the phosphorylation of all the secreted salivary proteins and peptides is not fully active during fetal life [86]. Interestingly, all these proteins are considered currently potential biomarkers of tumors with embryonic etiology in the adult. Indeed, these proteins and the interplay between different enzymes acting on them during fetal life might contribute to the molecular events responsible for cell growth and death. On the contrary, their abnormal regulation and expression in the adult might be at the basis of anomalous cellular growth and might be connected to the development of different neoplasms [87]. Indeed, cancer stem cells could have direct access to embryologic programs, including the capacity to synthetize and modify proteins and peptides normally secreted only during intrauterine life [88]. Specific analysis targeting these proteins or investigating the change of the activities of the enzymes involved in their processing could be useful for the early diagnosis of neoplastic events and should be better investigated in the next future. The level of the proteins characteristic of adult saliva increases after birth with different trends [86, 89], reaching the concentration detectable in the adult after the puberty (Figure 3). These dynamic changes of saliva during paediatric age have to be taken into account for the choice of proper controls in the studies performed to search biomarkers in paediatric diseases [85].

Metabolomic is another useful chance for salivary biomarkers discovery. A metabolomic study carried out with micro‐HPLC coupled to Q‐TOF MS on WS from 37 OSCC patients, 32 patients with oral leukoplakia, and 34 healthy subjects showed characteristic metabolic signatures for the three groups. After estimation of the predictive power, the authors established that valine, lactic acid, and phenylalanine in combination yielded satisfactory accuracy (0.89 and 0.97), sensitivity (86.5 and 94.6%), specificity (82.4 and 84.4%), and positive predictive value (81.6 and 87.5%) in distinguishing OSCC from controls and patients with oral leukoplakia, respectively [53]. Another metabolome profiling of human WS was carried out to search metabolite signatures of OSCC. Conductive polymer spray ionization mass spectrometry (CPSI‐MS) was employed to record a wide range of metabolite species within a few seconds, making this technique appealing as a point‐of‐care method for the early detection of OSCC. Saliva samples from 373 volunteers including 124 healthy controls, 124 premalignant lesions, and 125 OSCC patients, respectively, were collected for discovering and validating dysregulated metabolites and determining altered metabolic pathways. Metabolite markers were reconfirmed at the primary tissue level by desorption electrospray ionization MS imaging (DESI‐MSI), demonstrating the reliability of diagnoses based on saliva metabolomics. With the aid of machine learning (ML), OSCC and premalignant lesions can be distinguished from the normal physical condition in real time with an accuracy of 86.7%, on a person‐by‐person basis. These results suggest that the combination of CPSI‐MS and ML is a feasible tool for accurate, automated diagnosis of OSCC in clinical practice. [55]. Metabolomic studies of WS have been recently reviewed [55, 90–92].

Saliva contains exosomes, nanoparticles offering relevant chances for the detection of innovative protein biomarkers with a particular concern to different types of cancer [64, 93, 94]. Additionally, salivary exosomes have recently been revealed to contain potential biomarkers for the diagnosis of Sjögren's syndrome [95] and α‐synuclein, a potential biomarker of Parkinson's disease [67].

Analysis of microRNAs (miRNAs), which are small non‐coding and single‐stranded RNAs able to regulate gene expression post‐transcriptionally [60, 96], has been also performed, and various mi‐RNAs with diagnostic potential for head and neck squamous cell carcinoma have been recently detected in salivary exosomes too [61]. For instance, altered mi‐RNA patterns have been observed in patients with Sjögren's syndrome [62]. Specific salivary miRNAs could be promising biomarkers for autism spectrum disorders [97] and for age‐associated oculopharyngeal muscular dystrophy [63].

### 4.1 New analytical platforms

In addition to the different ‐omic platforms, the diagnostic potential of WS continues to be investigated using numerous conventional and unconventional techniques.

For instance, a simple analysis to determine the levels of salivary calcium ions by the colorimetric O‐cresolphthalein complexone method showed a very significant correlation with bone mineral density. In the post‐menopausal women, compared to the control group, a significant increase in salivary calcium level has been observed. Similarly, the study showed a negative correlation between salivary calcium and serum estrogen, substantiating the hypothesis that salivary calcium level could be a marker of osteoporosis in post‐menopausal group [98].

In the search for low‐cost and wearable analytical apparatus, various microfluidic paper‐based analytical devices for salivary analyses have been recently developed. One of them was able to determine the concentration of nitrate and nitrite in human WS [68]. A cellulose acetate‐coated mouthguard biosensor has been developed for in vivo salivary glucose measurements, which can be useful for a non‐invasive management of diabetic patients [69]. Shen and colleagues were able to arrange an origami electrical biosensor depositing by quantitative inkjet printing a pyrene carboxylic acid‐modified single‐walled nanotube on a paper substrate. Multiple antibodies were immobilized onto the paper surface for highly sensitive and specific field‐effect transistors chemiresistors biosensors able to detect human serum albumin and immunoglobulin G at the picomolar range in human WS [70]. This last study showed that the technology has the tools to develop sensing systems with multiple functions based on the use of inexpensive, semi‐automated, and easy‐to‐use one paper‐based microfluidic platforms.

The use of nanotechnologies using specific biosensor and bioelectronics for rapid, accurate wearable analyses of salivary specimens has been recently reviewed [71].

The availability of new spectroscopic methods allowing deep investigation of physical and chemical properties of biological fluids has recently extended the diagnostic applications of WS. For instance, the NMR quantification of 21 metabolite in patients with primary Sjögren's syndrome (pSS) was able to establish that salivary levels of choline, taurine, alanine, and glycine were significantly elevated with respect to controls. The authors concluded that the analysis could be useful for monitoring response to pharmacological treatment in pSS [66]. Surface‐enhanced Raman spectroscopy can carry out a quantitative detection of the main monosaccharides (glucose, fructose, and galactose) in WS using a displace‐and‐trap mechanism. Moreover, due to the use of multiple optical interference‐free (1800‐2200 cm^−1^) signal‐independent Raman probes, the detection range of this platform (0.125^−7^ mg/dL) perfectly covers physiological concentrations, enabling the quantitative detection of glucose and galactose in human salivary samples. [58]. Recent use of attenuated total reflection‐Fourier transform infrared (ATR‐FITR) spectroscopy analysis of WS highlighted spectroscopic bands at 1041, 1433‐1302.9 cm^−1^ with significantly higher intensities in breast cancer patients, than healthy controls. These spectroscopic signatures were able to discriminate breast cancer patients with sensitivity and specificity of 90 and 70%, respectively. Even though these values are not particularly high, the authors concluded that this ATR‐FITR analysis could be useful in screening investigations [65]. Similarly, Raman fingerprints of human WS have been used to search signals for the diagnosis of amyotrophic lateral sclerosis (ALS) [59]. In this study, Laser‐Raman spectroscopy was able to evidence specific salivary signatures which seemed to be related to ALS patients compared not only to controls, but also to Parkinson's disease patients, and Alzheimer's disease patients. These last two interesting studies lead to rely on the diagnosis to a spectroscopic signal, without the support of information on the molecular identity of the factors discriminating for the disease. Considering these drawbacks and the large polymorphism characteristic of human saliva, strong validations are required before a spectroscopic analysis can be accepted in clinical diagnostics.

Even though previsions are always difficult, the use of whole saliva for diagnostic purposes will surely increase in the coming years, and the hope is that it will allow the early diagnosis of local and systemic diseases. Salivary tests could offer the advantage of using simple and economical devices, thus allowing fast analyses, the results of which, if outside the normal range, could be substantiated by more precise and expensive determinations.

## CONFLICT OF INTEREST

The authors have declared no conflict of interest.
